# Mesenchymal Stem Cells Derived and Cultured from Glioblastoma Multiforme Increase Tregs, Downregulate Th17, and Induce the Tolerogenic Phenotype of Monocyte-Derived Cells

**DOI:** 10.1155/2019/6904638

**Published:** 2019-05-02

**Authors:** Kalina Tumangelova-Yuzeir, Emanuil Naydenov, Ekaterina Ivanova-Todorova, Ekaterina Krasimirova, Georgi Vasilev, Sevdalin Nachev, Dobroslav Kyurkchiev

**Affiliations:** ^1^Laboratory of Clinical Immunology, University Hospital “St. Ivan Rilski,” Department of Clinical Laboratory and Clinical Immunology, Medical University of Sofia, Sofia 1431, Bulgaria; ^2^Clinic of Neurosurgery, University Hospital “St. Ivan Rilski,” Medical University Sofia, 15 “Acad. Ivan Geshov” Str., 1431 Sofia, Bulgaria; ^3^Laboratory of Clinical Pathology, University Hospital “St. Ivan Rilski,” Medical University Sofia, 15 “Acad. Ivan Geshov” Str., 1431 Sofia, Bulgaria

## Abstract

Mesenchymal stem cells (MSCs) possess immunosuppressive properties and have been described in the tumor microenvironment of glioblastoma multiforme (GBM). This manuscript has two major topics—first, to describe isolated and cultured MSCs derived from GBM (GB-MSCs) and second, to examine their *in vitro* immunosuppressive capacity. Our results display cells with morphology and phenotype, clonogenic ability, and osteogenic potential, typical for MSCs. Furthermore, the cultured cells show intracellular expression of the neural markers Nestin and GFAP. They express PD-L1 and secrete TGF*β*, CCL-2, PGE2, IL-6, and sVEGF. Coculturing of GB-MSCs with PBMCs isolated from healthy donors results in a decreased percentage of Th17 lymphocytes and an increased percentage of Tregs. Regarding the impact of GB-MSCs on monocytes, we establish an augmented expression of CD14 and CD86 along with diminished expression of HLA-DR and CD80, which is associated with tolerogenic phenotype monocyte-derived cells. In conclusion, our results describe in detail GBM-derived and cultured cells that meet the criteria for MSCs but at the same time express Nestin and GFAP. GB-MSCs express and secrete suppressive molecules, influencing *in vitro* T cells and monocytes, and are probably another factor involved in the immune suppression exerted by GBM.

## 1. Introduction

Glioblastoma multiforme (GBM) is the most common malignant primary central nervous system (CNS) tumor in adults, with a median survival of about 12-14 months [1, 2]. Amongst the many hypotheses aiming to unravel the GBM carcinogenesis, there are two leading theories—the clonal evolution model and the cancer stem cell hypothesis. According to the clonal model, a single cell within a tumor progressively acquires competitively advantageous genetic mutations, leading to its uncontrolled proliferation [3]. According to the cancer stem cell hypothesis, cancer stem cells (CSCs) are tumorigenic “roots of cancer” which possess unlimited capacity for symmetric and asymmetric cell division [4]. CSCs are a small fraction of multipotent cells at the apex of a hierarchically organized cell population characterized by self-renewal capacity, a process at least partially controlled by epidermal growth factor (EGF) and beta fibroblast growth factor (bFGF). These mitogens operate through their receptor tyrosine kinases (RTKs) and provoke activation of downstream pathways such as the phosphoinositide 3-kinase/Akt (PI3K/Akt) and mitogen-activated protein kinase (MAPK). Of major importance for the maintenance of CSC self-renewal are Notch, TGF*β*, sonic hedgehog, and Wnt/*β*-catenin signaling pathways [5]. The self-renewal capacity is assessed by *in vitro* tumorsphere formation assay, where CSCs are *in vitro* cultured in serum-free medium containing EGF and bFGF [5]. CSCs are phenotypically characterized by the expression of certain markers amongst which of major importance are CD133, Nestin, Sox-2, CD44, and Oct-4 [6, 7]. Regarding the origin of CSCs, it has been assumed that they form through the transformation of neural stem cells (NSCs) located in subventricular and subgranular zones of the brain. This transformation can affect type B NSCs as well as the transit amplifying cells (type C) and even their more differentiated progeny [3]. The necrotic zones of GBM along with the perivascular GBM niche may serve as neurogenic niches for the forming CSCs. In these niches, CSCs communicate with a multitude of cells resulting in activation of the Notch signaling pathway which is responsible for the maintenance of CSC renewal [5].

At least two distinct groups of CSCs isolated from GBMs have been identified—proneural and mesenchymal types, as they use different signaling pathways and have a distinct mRNA profile [8]. A characteristic feature of the mesenchymal-type CSCs is CD133 negativity, association with aggressive tumor progression, and that it is regulated by aldehyde dehydrogenase and by the TGF*β* signaling pathway as well [9, 10].

There is a growing body of literature which suggests that CSCs are not the exclusive type of stem cells observed in GBM and turns the researcher's attention to the role of mesenchymal stem cells (MSCs) in the central nervous system. According to the classic definition, MSCs are fibroblast-like progenitor cells adhering to a plastic surface that possess the capacity to self-renew and can differentiate into several mesenchymal lineages [11]. Furthermore, MSCs exhibit potent immunosuppressive properties. MSCs have been described in almost all organs and tissues including CNS [12, 13]. According to some papers, pericytes in CNS, which cover more than 30% of the cerebral capillary surface, are in fact MSCs. There are evidence supporting this viewpoint—pericytes express MSC-specific markers, as well as they possess the ability to differentiate into osteogenic, adipogenic, and chondrogenic lineages [14].

Along with the tumorsphere formation assay mentioned before, another major approach is expanding GBM stem cell in adherent culture condition (serum containing). These two fundamental models, as well as various “intermediate models” models, have been previously discussed in our publications [15, 16]. Studies on GBM-cultured adherent cells suggest that they actually represent glioblastoma MSCs (hereinafter abbreviated as GB-MSCs). Moreover, even under tumorsphere culturing conditions, CD133-negative cells growing like adherent cells and manifesting clonogenic properties have been observed [17, 18]. The group of Nakahata describes that the adherent growing GBM cell lineage U87MG displays the mesenchymal phenotype and shares common features with MSCs. The cells from U87MG express typical MSC markers: CD44, CD90, CD105, CD73, and CD29 and are capable of adipogenic, chondrogenic, and osteogenic differentiation [19].

For the first time, the group of Hossain isolated, cultured and proved GB-MSCs, demonstrating that the majority of these cells are phenotypically and genetically distinct from CSCs. The authors isolated the cells using standard protocol for isolation and culturing of MSCs and ascertained that these cells are nontumorigenic and meet all the criteria for MSCs of the International Society for Cellular Therapy—typical morphology, adherent growth, positive expression of CD90, CD73, and CD105, lack of expression of CD45 and CD34, and the ability to differentiate into adipogenic, osteogenic, and chondrogenic lineages [20]. Furthermore, the authors describe the same cytokine secretion as typical for MSCs IL-6, IL-8, CCL-2, and Gro-*α* [21].

The aim of the present study was to isolate, culture, and characterize GB-MSCs. We also aimed to investigate their cytokine secretion, the expression of PD-L1, and their immunoregulatory activity exerted on T cells and monocytes when GB-MSCs are cocultured with PBMC and PBMCs are cultured with GB-MSC supernatants. We describe in detail GBM-derived and cultured cells that meet the criteria for MSCs but at the same time express Nestin and GFAP. They express and secrete suppressive molecules, influencing *in vitro* T cells and monocytes, and are probably another factor involved in the immune suppression exerted by GBM.

## 2. Materials and Methods

### 2.1. Subjects and Sample Collection

Ten patients (seven women and three men aged 50-76 years) diagnosed with GBM were recruited sequentially in the study. Tissue sample (1-3 cm^3^) from each patient was collected during resection of the tumor in the Clinic of Neurosurgery after signing the informed consent in agreement with the Ethics Committee of the University Hospital “St. Ivan Rilski,” Sofia. Tissue samples were taken from a precisely defined area of the tumor referred to as a “viable” area [22]. The samples were immediately placed in sterile phosphate-buffered saline (PBS) (pH 7.4) and delivered to the laboratory within 30 minutes.

For the purpose of isolating and culturing of peripheral blood mononuclear cells (PBMCs), ten healthy volunteers (eight women and two men, aged 30 – 50 years) were also sequentially included in the study. Eight milliliters of peripheral venous blood was collected to isolate PBMCs using BD Vacutainer CPT (NC: 1 ml, Ficoll: 2 ml) (REF 362782, BD, USA). The cells were gradient centrifuged, separated, washed, and adjusted at a concentration of 1.10^5^ cells per well. Healthy volunteers also have signed the informed consent.

### 2.2. Isolation and Culturing of GB-MSCs

Isolation and culturing of adherent GBM-derived MSCs (GB-MSCs) were described in detail previously [15]. In the present experiments, the cells were cultured in DMEM/F-12 medium (PAN-Biotech, Germany), containing 20 ng/ml bFGF (Abcam, UK), 10% (*v*/*v*) fetal bovine serum (FBS) (PAA Laboratories), and antibiotic/antimycotic 100 IU/l (PAA Laboratories, Austria). After 10-14 days, they formed monolayer-consisting fibroblast-like cells.

### 2.3. Clonogenic Cell Growth of GB-MSCs

The monolayer of GB-MSCs at first passage was trypsinized with 0.05% trypsin/0.02% EDTA (PAN-Biotech, Germany) and centrifuged for 10 minutes at 280 g. The cells were washed with PBS, and the cell pellet was resuspended in 2 ml of DMEM/F-12 in the presence of 10% FBS culture medium. The cells were counted in a Bürker chamber and placed in a 25 cm^2^ PVC plate (SPL Life Sciences, Korea) at a concentration of 400 cells/cm^2^. Microscopic observation of the culture was performed daily to trace the origin of each single-cell clone. After two weeks of culturing, the formed GB-MSC colonies were washed twice with ice-cold saline solution. The cells were fixed with cold undiluted methanol for 15 minutes at 4°C and subsequently stained with 0.5% (*w*/*v*) crystal violet in 25% (*v*/*v*) for 10 minutes at room temperature. The stained cell colonies from GB-MSCs were used to determine clonogenic effectiveness. Colonies consisting of at least 20 cells were counted (in triplicate), and clonogenic effectiveness (CE) was calculated by the following formula:

(1) $$\begin{array}{c} \text{CE }\left[\%\right]=\frac{\text{number of count colonies}}{\text{number of sown cells}}\times 100. \end{array}$$

### 2.4. Osteogenic Differentiation of GB-MSCs

GB-MSCs at above 80% confluency at the first passage were plated in 24-well plates (2 cm^2^, SPL Life Sciences, Korea) at a concentration of 5 × 10^4^ cells/cm^2^ and cultured in DMEM/F12 cell medium in the presence of 10% FBS and the following differentiation factors: 100 nM dexamethasone (Sigma-Aldrich),0.2 mM ascorbic acid-2-phosphate (Sigma-Aldrich), and 10 mM *β*-glycerophosphate (Sigma-Aldrich). Fresh osteogenic culture medium was added every 72 hours for 4 weeks. In parallel, control cells were cultured in medium without differentiating factors. The degree of osteogenic differentiation was determined as follows:

1. By applying a colorimetric assay for assessment of alkaline phosphatase activity, GB-MSCs were washed with PBS and 150 *μ*l of alkaline phosphatase buffer was added to each well (0.05 M Na_2_CO_3_, 0.5 mM MgCl_2_, pH = 9.5) containing 0.1% (*v*/*v*) Triton X-100 (Merck, Germany). The plate was frozen at -80°C for 5 min and then immediately thawed. This procedure was carried out three times. 150 *μ*l of the alkaline phosphatase substrate solution of 4-p-nitrophenylphosphate (3.5 mM in alkaline phosphatase buffer) was added to the wells with already-lysed cells, and the resulting color reaction was read spectrophotometrically on a micro-ELISA reader (Dynatech AG, USA) at a wavelength of 405 nm
2. By specific histological staining to demonstrate the deposition of Ca^2+^ in the extracellular matrix (von Kossa and alizarin red staining), the osteogenically differentiated cells were washed once with PBS and then treated with 1% (*w*/*v*) silver nitrate solution (AgNO_3_, Sigma-Aldrich, USA) under ultraviolet light (*λ* = 366 nm, CAMAG Reprostar 3 transluminer, Switzerland) for 60 min. The presence of Ca^2+^ deposits with characteristic black color was detected by inverted light microscopy (MICROS, Austria)

Staining of GB-MSCs with alizarin red was also done. After washing with PBS once, the cells were fixed with 10% (*v*/*v*) neutral formalin (Merck, Germany) for 30 minutes at room temperature, followed by washing with distilled water and staining with 2% (*w*/*v*) solution of alizarin red S (Sigma-Aldrich, USA) for 40 minutes. In the presence of Ca^2+^ deposits, a bright red color is observed. In the absence of calcium deposits, the staining is pale yellow

### 2.5. Coculturing of GB-MSCs and Media from GB-MSCs with Human PBMCs

The “ideal” approach should be to coculture GB-MSCs with matched PBMCs aiming to exclude MHC nonmatched allo response. Our original idea was to do so; however, we faced key technical issues. GB-MCSs have to be isolated immediately after the surgical intervention, and a certain time for their cultivation is required. It turned out extremely laborious to find an eligible MHC-matched healthy donor in this narrow time frame or alternatively to coordinate the second visit of the patient with the time of PBMC's isolation.

Upon reaching a confluency of more than 80% of each GB-MSC culture, at the 1st passage, the cell culture media was collected sterile at the 72nd hour, centrifuged at 280 g for 10 min, and transferred to a sterile well. PBMCs at a concentration of 1.10^5^ cells per well were added to the following:

1. GB-MSC-adherent cell culture
2. 72-hour medium of the same GB-MSCs
3. An empty well as a control, where there was only DMEM/F-12 medium containing 10% FBS bFGF and antibiotic/antimycotic

All cells were cultured under standard conditions (37°C, 5% CO_2_, and 95% humidity) for 72 hours, and then PBMCs from all wells were gently resuspended and separated with the culture medium, centrifuged at 280 g for 10 min, washed, and analyzed by flow cytometry. GB-MSCs were washed with sterile PBS twice, trypsinized, and prepared for flow cytometric analysis.

### 2.6. Flow Cytometry Analysis

The trypsinized GB-MSCs and their respective PBMCs were washed with PBS, centrifuged at 280 g for 10 minutes, counted, and brought to a concentration of 1.10^5^ cells. GB-MSCs were tested for expression of the following surface markers: PD-L1-APC (BD Pharmingen, USA), CD73-PE, CD90-FITC, CD105-PerCP/Cy5-5 (eBioscience, USA), CD45-FITC/CD34-PE, CD44-FITC, CD146-PE, and HLA-A, B, C-FITC (Becton Dickinson, USA) and intracellular markers: Nestin-PE, Sox-2-PerCP, and GFAP-Alexa Fluor 488 (eBioscience, USA). For the detection of intracellularly expressed markers, the Cytofix/Cytoperm Fixation/Permeabilization kit (BD Pharmingen, USA) was used following the manufacturer's instructions.

PBMCs from healthy volunteers were tested for surface expression of CD3-FITC, CD4-PerCP, CD161-PE, CD196-Alexa Fluor 488, CD25-FITC, CD14-FITC, CD80-PE, CD86-APC, and HLA-DR-PerCP and intracellular expression of FoxP3-PE (BD Pharmingen, USA) by using the Cytofix/Cytoperm Fixation/Permeabilization kit (BD Pharmingen, USA). Cells were processed according to the manufacturer's instructions, fixed with CellFix (BD, USA), and analyzed by FACSCalibur flow cytometer (BD, USA). Software CellQuest and WinMDI 2 were used for further analysis.

### 2.7. Immunoenzyme Assay (ELISA)

72-hour culture media from ten GB-MSC culture which reached over 80% confluency were tested for the presence of IL-4, IL-6, IL-8, IL-10, IL-12, IL-17A, IL-18, IL-23, TGF*β*1, IFN*γ*, TNF*α*, CCL-2, VEGF*α*, sPECAM, sICAM (Gen-Probe Diaclone SAS, France), and PGE2 (Abcam, UK) strictly following the manufacturer's instructions.

### 2.8. Statistical Methods

For the statistical processing of the data obtained, the statistics package SPSS v. 21 (IBM) and GraphPad Prism 7 were used. Differences were considered as significant at *p* < 0.05.

## 3. Results and Discussion

### 3.1. GB-MSC Morphology, Clonogenicity, Osteogenic Differentiation, Marker Expression, and Cytokine Secretion

**Figure 1:**
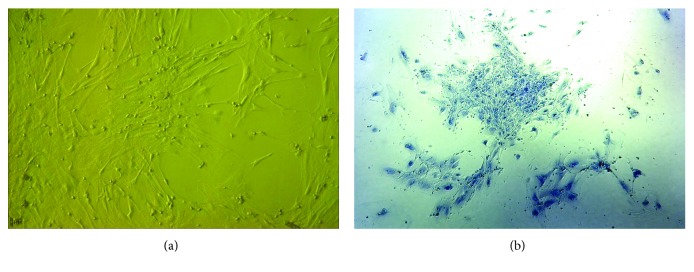
Morphology and clonogenicity of GB-MSCs. GB-MSCs demonstrate adherent growth and fibroblast-like morphology typical for “classical” MSCs (a). GB-MSCs demonstrated clonogenic capacity by colony formation. Crystal violet staining (b).

**Figure 2:**
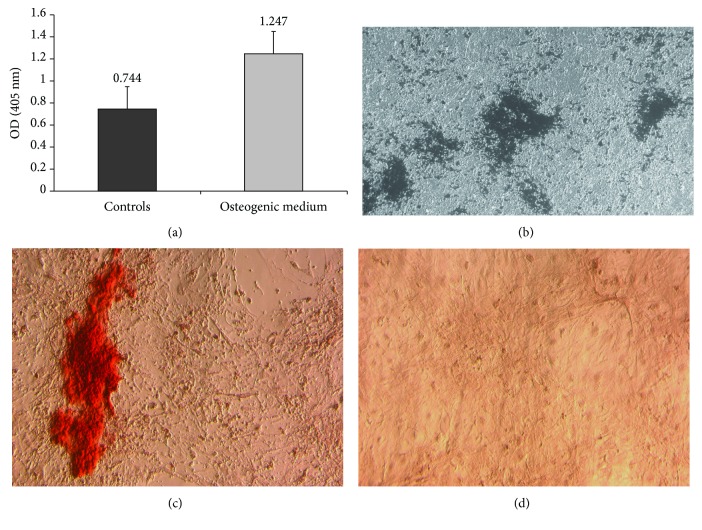
Differentiation of GB-MSCs in osteogenic direction. Proven by quantification of alkaline phosphatase activity (a), von Kossa (b), and alizarin red staining (c). Control cells cultured in noninductive media (d).

Cells from the GBM “viable” region were isolated and cultured with bFGF and 10% FBS. The cells grew fibroblast-like, adhering to the bottom of the plate and forming a monolayer similar to that of “classical” MSCs after 10-14 days of culturing (Figure 1(a)). GB-MSCs demonstrated clonogenicity forming clearly identifiable colonies when the cell cultures were stained with crystal violet (Figure 1(b)). MSCs are characterized by their ability to differentiate into the osteogenic direction. At the end of the osteogenic differentiation experiments, elevated alkaline phosphatase activity of our cells was detected (Figure 2(a)) compared to corresponding control cell cultures. The presence of Ca^2+^ crystals was demonstrated by von Kossa (Figure 2(b)) and alizarin red (Figure 2(c)) staining.

**Figure 3:**
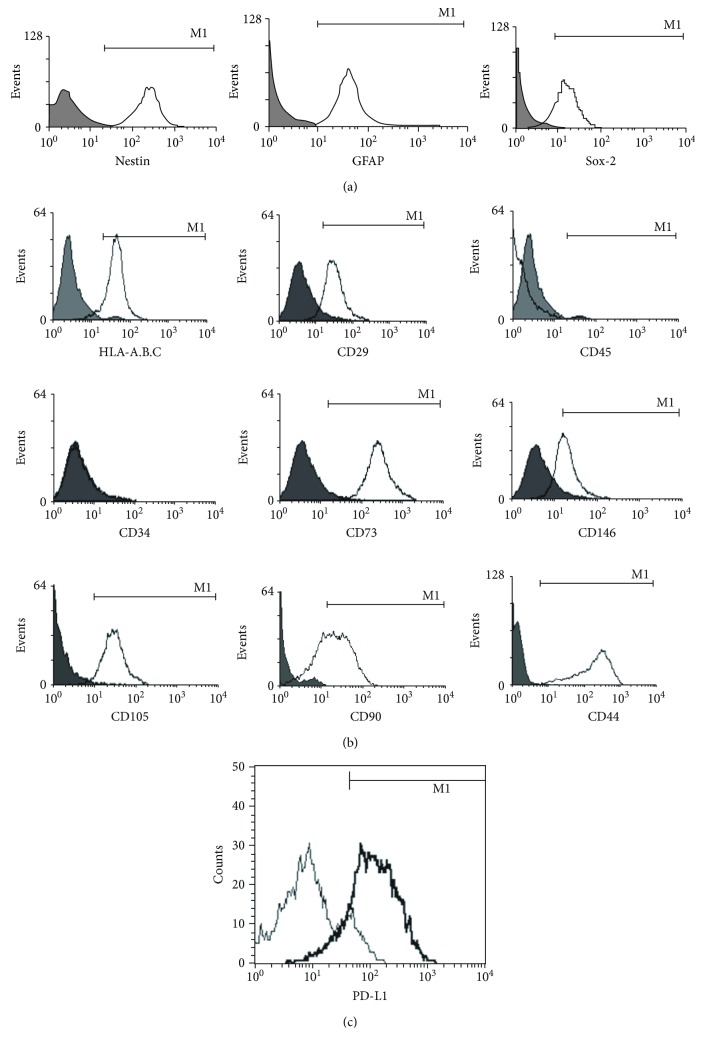
Marker expression by GB-MSCs. GB-MSCs have positive expression for Nestin (mean 97.1%), Sox-2 (mean 79.3%), and GFAP (mean 71.3%) (a). GB-MSCs express characteristics typical for “classical” MSC markers. More than 90% of the cells expressed CD73, CD90, CD105, CD29, CD44, CD146, and HLA-A, B, C but not CD34 and CD45 (b). GB-MSCs express PD-L1. On average, 73.2% of the cells in the cell cultures expressed this marker (c).

GB-MSC cultures were examined by flow cytometric assay for expression of some markers associated with CSCs—Nestin, GFAP, and Sox-2. Each marker was expressed strongly by GB-MSC cultures, with an average of 97.1% (95.3% - 99.8%) of the cells expressing Nestin, 79.3% (65.8% - 84.7%) expressing Sox-2, and 71.3% (68.2%-73.3%) expressing GFAP (Figure 3(a)).

According to the Minimum Criteria of ISCT (International Society for Cellular Therapy), to identify cells as MSCs, they should express CD73, CD105, and CD90 markers on their surface but not markers for hematopoietic cells—CD45 and CD34. GB-MSCs showed positive expression for CD73, CD90, CD105, CD29, CD146, and HLA-A, B, C by at least 90% of the cells and lack of expression of CD34 and CD45 (Figure 3(b)). CD44 which is believed to be expressed by both CSCs and MSCs was expressed in 78.8% (72.8% - 80.5%) of GB-MSCs.

All cell cultures showed positive expression for PD-L1, with an average of 73.2% (63.3%-78.7%) of the cells expressing this marker (Figure 3(c)). Sixteen cytokines were tested and the concentrations of IL-6, IL-8, IL-17A, TGF*β*1, CCL-2, PGE2, and sVEGF-*α* were obtained (Table 1).

Our results raise the question about the nature of the cultured cells. Tumors, including GBM, possess the ability to attract MSCs [23], and GB-MSCs represent an important part of the CSC cellular niche. They can be attracted to the tumor and may enhance tumorigenicity, proliferation, and self-renewal capacity of CSCs [21]. In addition, there is evidence that GB-MSCs into the tumor microenvironment can be a product of CSC transdifferentiation. It has been shown that CSCs are associated to the process of epithelial to mesenchymal transition, and it is likely that they undergo a proneural-mesenchymal shift. Transcription factors c/EBP13, STAT-3, and MLK4 play a key role in this process [24, 25]. It has been also found that CSCs are genetically similar to both NSCs and MSCs, and their ability to transdifferentiate into pericytes/MSCs has been described [4, 14]. Hossain et al. proved that along with the classical bone marrow-derived MSCs attracted to the tumor microenvironment, there also exist MSCs with genetic alterations typical for CSCs (deletions in chromosome 10 and amplification in chromosome 7) [21].

It is quite important to also note that not only CSCs can transdifferentiate into GB-MSCs; likewise, a possible scenario is the one with MSC neuronal transdifferentiation. It is known that MSCs dispose of wide assortment of neural genes. The group of Blondheim found that MSCs can express 12 neural genes, 8 neuro-dopaminergic system-related genes, and 11 gene-encoding transcription factors [26]. MSCs can also spontaneously express Nestin, NeuN, and *β*III tubulin—phenotypic markers associated with the neural tissue [27]. At the same time, MSCs retain their characteristic morphology, typical markers, and ability to differentiate in mesenchymal cell lineages [26]. At least, *in vitro* MSCs are capable to transdifferentiate into neural and glial cells. Although this statement is a matter of debate for many types of MSCs, there is the notion that MSCs/pericytes in the CNS possess an increased capacity to transdifferentiate [14]. Particularly for GBM, an interesting hypothesis postulates that a central role in its development plays the so-called “cancer pericytes” or “cancer MSCs.” According to this assumption, “cancer MSCs” detach from the basal lamina of the vessels and migrate into the parenchyma where under the influence of specific conditions, they transdifferentiate into CSCs. Proliferation of the newly created CSCs leads to hypoxia and neoangiogenesis. This, in turn, leads to shift of the established conditions and to formation of mesenchymal phenotype CSCs which can turn into GB-MSCs, forming a “vicious cycle” [14].

It is well known that the tumor modifies its stromal environment in order to favor its development. From this point of view, the surrounding cells are often altered—a process known as “stromal corruption” [28]. Particularly good examples in such a context, affecting MSCs, are the so-called cancer-associated fibroblasts (CAF). In breast cancer, MSCs are attracted to the tumor and under its influence are modified in collagen-producing cells or CAFs [23]. Evidence to sustain the assertion of “stromal corruption” affecting MSCs is the data reporting that whereas parts of GB-MSCs are classical bone marrow-derived MSCs, other GB-MSCs possess the characteristic genetic features of CSCs. It has also been suggested that the latter cells are a result of the transdifferentiation of CSCs. There is a third group of GB_MSCs whose genetic features are not typical for either normal MSCs or CSCs [21]. Exactly, these kinds of cells are probably former classical MSCs transformed into GB-MSCs as a result of “stromal corruption.”

The results of our experiments have shown that the isolated and cultured cells are equivalent to MSCs regarding their adherent growth, typical fibroblast-like morphology, clonogenicity, expression of phenotypic markers, and osteogenic differentiation capacity. Moreover, we have detected that they express intracellularly neural markers such as Nestin and GFAP. If we take into account the aforementioned data and the literature-based evidence, the following scenarios might apply:

- The obtained GB-MSCs could be MSCs attracted to the tumor and induced to neuronal transdifferentiation
- The obtained GB-MSCs could be a result of the transdifferentiation of CSCs into MSCs
- GB-MSCs might be classical MSCs (or MSCs affected by “stromal corruption”), taking into consideration that MSCs can spontaneously express Nestin [14, 26] and GFAP [29]
- The cell culturing process might be responsible for the expression of Nestin and GFAP. When we talk about *in vitro* transdifferentiation of MSCs to neural cells, the puzzling question of the influence of the culture medium could not be avoided. The main issue with the expression of Nestin and GFAP by GB-MSCs, as markers of their transdifferentiation, is that too many agents could induce it. The vast diversity of these factors, their rapid action, and the reversibility of the process raise the question to what extent is it a true transdifferentiation [30]

### 3.2. Effect of GB-MSCs on Treg Cells in the Pool of PBMCs

The GB-MSCs that we obtained express PD-L1 and secrete the same cytokines as MSCs isolated from other tissues and described by other groups as well as by our team [31]. These cytokines, as PD-L1, are generally associated with immunosuppressive action on immune-competent cells. Our results have demonstrated that the number of Tregs (CD4+CD25+FoxP3+) increases under the influence of GB-MSCs both in coculturing and under the action of GB-MSC medium.

**Figure 4:**
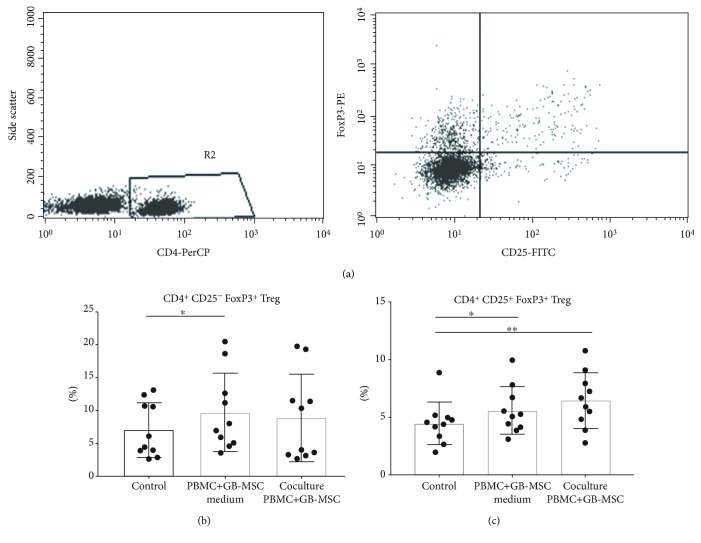
GB-MSCs lead to increased percentage of Tregs. Representative dot plot analysis of FoxP3+ T helper cells in the pool of PBMCs (a). Distribution of CD4+CD25-FoxP3+ T helper cells in the pool of the control PBMCs, compared to those cultured with the supernatants from GB-MSCs (*p* = 0.007) and cocultured with GB-MSCs (b). Distribution of CD4+CD25+FoxP3+Treg cells in the pool of the control PBMCs compared to those cultured with the supernatants from GB-MSCs and cocultured with GB-MSCs; *p* = 0.028 and *p* = 0.009, respectively (c).

The presence of Treg cells and the effect of GB-MSCs on them were investigated by flow cytometry based on the expression of markers typical for Tregs—CD4, CD25, and FoxP3. Tregs were detected as a percentage of all CD4+ T cells from the studied PBMCs (Figure 4(a)). The effect of the supernatants of GB-MSC culture and the cellular contact of GB-MSCs on Tregs was compared to control cells cultured with DMEM/F-12, bFGF, and 10% FBS medium and grown under the same conditions. FoxP3-positive T helper cells were divided into two subpopulations based on the CD25 expression: CD4+CD25-FoxP3+ and CD4+CD25+FoxP3+ cells. CD4+CD25-FoxP3+ cells were about 7.08% (2.65% -13.3%) of the T helper population in the control PBMCs. We found a significant increase in the percentages of the latter under the influence of GB-MSC supernatants as follows: 9.73% (3.57% - 20.5%, *p* = 0.007). Regarding the cellular contact with GB-MSCs, CD4+CD25-FoxP3+ cells showed a certain trend toward significance: 8.91% (2.69% - 19.7%) (Figure 4(b)). The classical Treg subpopulation CD4+CD25+FoxP3+ were 4.49% (1.99% - 8.87%) of the control PBMCs, 5.59% (3.11% - 9.95%, *p* = 0.028) when cultured with the supernatants from GB-MSCs, and 6.46% (2.80% -10.77%, *p* = 0.009) when cocultured with GB-MSCs (Figure 4(c)). From the results obtained, it can be concluded that under the influence of GBМ-MSC medium, there was a significant increase in both CD4+CD25-FoxP3+ T helper population and CD4+CD25+FoxP3+ Treg population. Coculturing with GB-MSCs, CD4+CD25-FoxP3+ showed a tendency to increase, whereas for CD4+CD25+FoxP3+, a significant increase was found.

A high percentage of T regulatory cells (Tregs) is present in both peripheral blood and tumor microenvironment of GBM (similarly to other tumors). It is considered that CCL-2 and IDO, secreted by glial cells, play a major role in attracting Tregs into the tumor microenvironment [32–38]. There is evidence showing that the number of Tregs correlates with a higher degree of GBM malignancy and with shorter survival of the patients [39–41].

The increased Tregs under the influence of GB-MSCs in our experiments raise the question of whether it is due to expansion of already-available Tregs or it is a result of the conversion of normal T lymphocytes into inducible Tregs (iTregs). There is evidence in the literature supporting each of both assumptions. Some authors conclude that the main mechanism leading to an increased number of Tregs in GBM includes the migration and proliferation of Tregs under the influence of CCL-22 and CCL-2 [38, 42]. In accordance with this data is the increased expression of CCR-4 (which is the CCL-2 receptor) on Tregs in GBM [34, 41].

On the other hand, TGF*β* can induce the formation of iTregs in gliomas [38, 43]. If GB-MSCs are considered to be classical MSCs, their ability to induce T cell conversion into iTregs has been repeatedly described by other authors as well as by us [44–46]. A number of factors, both membrane-bound and secreted, are involved in this process. Both the membrane-bound and the secretory TGF*β* [47–49], and PGE2 [50], play a central role in the MSC-induced T cell conversion into iTregs. Additionally, sHLA-G5 [51], IDO [52], and PGE2 in cooperation with IL-6 [53] take part in this process. Besides these cytokines, the interaction PD-L1/PD-1 plays a pivotal role in the conversion toward Tregs [54, 55]. The expression of PD-L1 has been demonstrated in glioma cell lines [56], “glioma stem-like cells” [35], and MSCs as well [57, 58]. It is also known that PD-L1 can perform its functions through its soluble form described in MSCs [58].

Our results could not give a definite answer if the increased Tregs are due to the proliferation of the already-available Tregs or they are a result of the T cell differentiation toward iTregs. However, we assume that the second scenario is more likely to occur. The reasons for this assumption are several. First, in our experiments, GB-MSCs express and secrete some of the factors related to the conversion into the iTreg direction: PD-L1, TGF*β*, and PGE2. Secondly, our results have shown a reduced number of Th17, taking into account that the conversion of Th17 in Tregs is a well-known fact [59]. Lastly, we observed an increase in CD4+CD25-FoxP3+ cells under the influence of GB-MSCs. This population, defined as “mysterious” cell population [60], in which the suppressive role is still a matter of debate [61, 62], is probably a peripheral reservoir for the formation of the “classical” Tregs [63]. The “infectious tolerance” pathway, described in the past, also supports the hypothesis for the creation of iTregs [64]. More recent studies confirm this process reflecting the T suppressor cell's capacity to generate new suppressors from the pool of conventional T cells [65, 66]. It has been described that iTregs, generated under the influence of TGF*β*, in turn, secrete TGF*β* where they “teach” conventional T cells, transforming them into iTregs [67]. Based on this statement, in our case, we speculate that TGF*β*, secreted by GB-MSCs, induce the creation of iTregs. By turns, iTregs induce the conventional T cells to differentiate to CD4+CD25-FoxP3+ and the latter in turn generate the classical Tregs (CD4+CD25+FoxP3+).

However, our results cannot give a definite answer whether the increased Tregs are proliferating nTregs or they represent iTregs generated from the conventional T lymphocytes. Further experiments are required to answer accurately. From that point of view, whether they are iTregs or proliferating nTregs is a substantial matter indeed; however, it is of secondary importance for the aims of the current study. In both cases, GB-MSCs exert indirect immunosuppression through an increase of Tregs—a key process for the survival of the tumor.

### 3.3. Effect of GB-MSCs on Th17 Cells in the Pool of PBMCs

**Figure 5:**
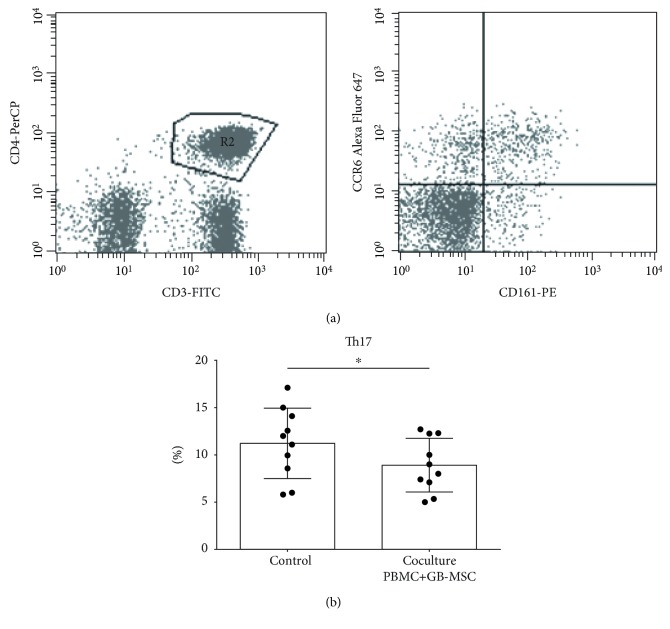
GB-MSCs downregulate the percentage of Th17 lymphocytes. Representative dot plot analysis of Th17 cells in the pool of PBMCs (a). The percentage of Th17 cells in control PBMCs, compared to those cocultured with GB-MSCs, *p* = 0.028 (b).

The influence of GB-MSC secretory factors and cellular contact on Th17 cells contained in the PBMCs isolated from healthy donors was reported based on the expression of markers typical for Th17 cells—CD3, CD4, CD161, and CD196. The results are presented as percentage from all CD4+ T cells (Figure 5(a)). The flow cytometric analysis of the data showed a lack of influence of the supernatant from GB-MSCs on Th17 cells (data not shown), where the percentage of Th17 cells in control PBMC was about 11.25% (5.80%-17.10%) of the T helper population and 11.55% (5.38%-17.23%) in PBMCs cultured with the supernatant. However, the experiment of coculturing of PBMCs from healthy donors with GB-MSC cultures showed a significant reduction in the percentage of Th17 cells to 8.81% (5.34%-12.70%) of the T helper population in PBMCs (*p* = 0.028) (Figure 5(b)).

Th17 are a lymphocyte subpopulation described in human gliomas as well as in mouse models, and there is evidence that dendritic cell vaccines using GBM antigens induce a Th17 immune response [68, 69].

Different approaches are used in the literature for identification of Th17 cells. The intracellular expression of IL-17, along with the cell surface markers, is the most accurate way to determine Th17 cells. However, our approach, albeit indirect, is often used in scientific papers to define Th17 subpopulation.

In addition, it has been shown that IL-17R can be expressed by CSCs and IL-17 stimulates their self-renewal capacity [70]. As it is well known, iTregs and Th17 share common features, and respectively, the formation of one or the other cell subpopulation is highly dependent on the cytokine ratio between the levels of TGF*β* and IL-6 [70, 71]. This also determines the development of cells bearing the features of both subpopulations, e.g., cells expressing FoxP3 and secreting IL-17 at the same time [72]. Th17 and Tregs synchronously increase simultaneously with the development of GBM, as Tregs are able to influence the Th17 cells [73].

With regard to Th17 subpopulation, our results have revealed a decrease in their number only when cocultured with GB-MSCs yet not under the influence of their medium. The probable mechanism has been described by Ghannam et al. who proved that MSCs transform Th17 cells into Tregs by effectuating a direct cell contact in them via CD54-CD11a/CD18 and CCL20-CCR6, resulting in PGE2 secretion and trimethylation at K4me3 of histone H3 in the FoxP3 gene locus promoter. At the same time, trimethylation of the RORC gene is suppressed and the former Th17 cell acquires the iTreg phenotype [74].

### 3.4. Effect of GB-MSC on Monocytes in the Pool of PBMCs

**Figure 6:**
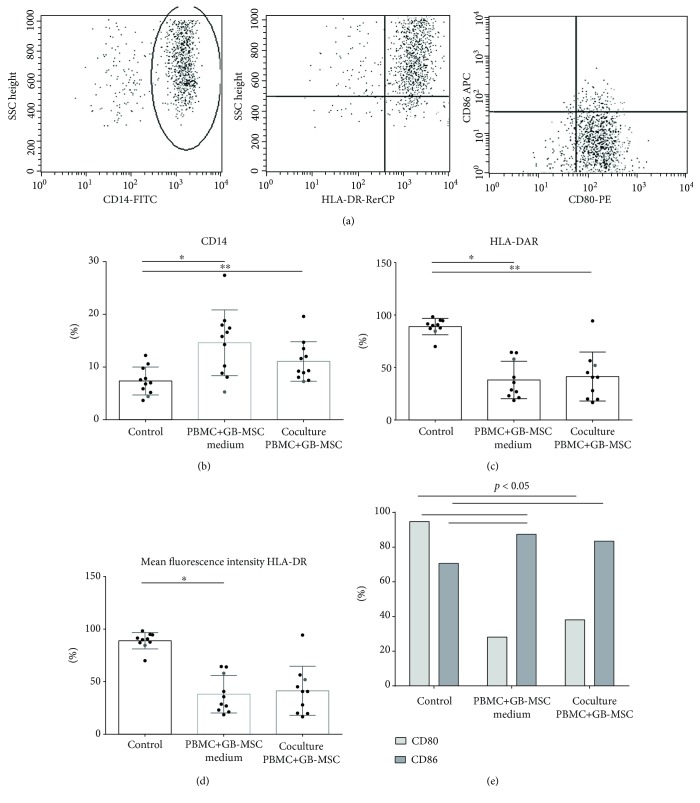
GB-MSCs induce tolerogenic phenotype monocyte-derived cells. Representative dot plot analysis of monocyte-derived cell detection in the pool of PBMCs (a). Comparison of the percentage of CD14-positive monocyte-derived cells in the composition of control PBMCs, PBMCs cultured with the supernatant from GB-MSCs (*p* = 0.005), and PBMCs cocultured with GB-MSCs (*p* = 0.007) (b). Comparison of the percentage of monocyte-derived cells expressing HLA-DR in monocyte-derived cells in the pool of control PBMCs and PBMCs cultured with supernatants of GB-MSCs (*p* = 0.005) and PBMCs cocultured with GB-MSCs (*p* = 0.005) (c). Comparison of the HLA-DR mean fluorescent intensity (MFI) in monocyte-derived cells in the pool of control PBMCs and PBMCs cultured with supernatants of GB-MSCs (*p* = 0.011) and PBMCs cocultured with GB-MSCs (d). Comparison of the percentage of CD80- and CD86-expressing monocyte-derived cells in the composition of control PBMCs and PBMCs cultured with the supernatant from GB-MSCs (*p* = 0.028 and *p* = 0.028, respectively) and PBMCs cocultured with GB-MSCs (*p* = 0.028 and *p* = 0.028, respectively) (e).

The flow cytometric analysis of monocytes in the PBMC composition isolated from healthy donors was performed based on the expression of the following markers: CD14, CD80, CD86, and HLA-DR (Figure 6(a)). Our results showed a significant increase in CD14+ cells compared to control cells in both experimental settings. An average of 7.65% (3.66%-12.20%) of CD14+ cells in control PBMCs, 15.53% (8.07%-27.4%, *p* = 0.005) CD14+ cells cultured with GB-MSC supernatant, and 11.44% (7.47%-19.6%, *p* = 0.007) CD14+ cells cocultured with GB-MSCs were found (Figure 6(b)). Expression of HLA-DR on CD14+ monocytes was examined, with a significant decrease of the percentage as well as of mean fluorescence intensity (MFI) on the cells. Control PBMCs showed that 88.94% (69% -98.3%) of CD14+ monocytes expressed HLA-DR, with a mean MFI of 1940.75 (86.60-4446.49). In PBMCs cultured with the supernatants from GB-MSCs, expression of this marker declined significantly—38.12% (18.57%-64.40%, *p* = 0.005) with MFI 1071.19 (136.34-2693.10, *p* = 0.011). PBMCs cocultured with GB-MSCs showed a significant decrease in the percentage of monocytes expressing HLA-DR compared to control cells—41% (16.60%-94.37%, *p* = 0.005). A decrease was also observed in the MFI—1497.40 (120.19-4171.25)—compared to the control monocytes without reaching significance (Figures 6(c) and 6(d)).

The effect of GB-MSCs on the expression of CD80 (B7-1) and CD86 (B7-2) molecules comprising the major costimulatory B7 complex was also investigated. In control PBMCs, 95.35% (85.9% -98.2%) expression of CD80 and 70.63% (21% -94%) of CD86 expression by monocytes were observed. The dynamics in the expression of B7 in both experimental settings were similar—there was a strong decrease in CD80 expression and an increase in CD86 expression compared to control monocytes. The mean expression of CD80 in culture supernatants was observed in 36% (16.8%-75.4%, *p* = 0.028) of CD14+ cells and that of CD86 was in 87.40% (72% -97.1%, *p* = 0.028). In coculturing with GB-MSCs, we obtained expression of CD80 being 38.07% (18.1% -66.9%, *p* = 0.028) and that of CD86 being 83.43% (72.8%-95.6%, *p* = 0.028) (Figure 6(e)).

Glioma-infiltrating monocytes have been described in GBM, and many authors have reported that they are attracted under the influence of CCL-2 and IL-8 [41, 75–77]. Typically, the tumor microenvironment induces alterations in the phenotype and the function of the monocytes and they differentiate eventually into various suppressor monocyte-derived cells such as M2-polarized tumor-associated macrophages [77], Tie-2-expressing monocytes, and myeloid-derived suppressor cells (MDSCs) [76, 78]. The common feature of these monocyte-derived subpopulations, besides their immunosuppressive properties, is their altered self-surface marker expression.

In GBM, the peripheral monocytes [79, 80] and their progeny that infiltrate the tumor display a reduced HLA-II and B7 complex (CD80/86) expression [75, 81] and secrete immunosuppressive mediators such as IL-10, arginase-1, PGE2, TGF*β*, sPD-1L, and sFAS-L [76]. All these factors determine their impaired antigen-presenting ability and their increased apoptosis-inducing capacity on the activated T lymphocytes [78]. It is believed that secretory factors, described as being secreted by CSCs and by glioblastoma cell lines [82], play a pivotal role for the generation of these monocyte-derived cells [83]. The major secretory factors, discussed in the literature and related to the monocyte “shift” in GBM, are TGF*β* [79, 82], IL-6 [82], and PGE2 [82, 83].

Our results have revealed that under the influence of both GB-MSC medium and upon a direct contact with PBMCs, an increased number of CD14-expressing cells has been observed. On the other hand, the number of CD14+ cells, expressing HLA-DR and CD80, has markedly decreased. We have also detected a reduction in the HLA-DR expression quantified by mean fluorescence intensity. At the expense of this, an increase of CD14+-expressing CD86 cells has been shown. Hence, GB-MSCs have induced an increased number of CD14+ monocyte-derived cells with reduced HLA-DR and CD80 expression, while at the same time, CD86 expression has raised. Thus, further experiments are required to determine the exact cell subpopulation derived from the former blood monocytes under the influence of GB-MSC. Along with dendritic cells, macrophages, MDSCs, and Tie-2, monocytes can as well differentiate in endothelial progenitors like in meso- and neuroectoderm cells [84].

While our results for CD14 and HLA-DR are consistent with the literature data, both for the effect of GBM lines [75, 81] and for that of MSCs [85–88], the results obtained for CD86 have surprised us, as most of the data have shown diminished expression of CD86. As far as we know, until the present moment, there is no evidence in the literature of the effect of GB-MSCs on CD86 monocyte expression. In all cases, the increase of CD86 along with the decrease of CD80 alters the structure of the B7 receptor, which would affect their antigen-presenting performance.

The described effects on monocyte-derived cells could be associated with the increased number of Tregs. It is well known that Tregs via the CTLA-4 receptor can bind to the B7 complex (CD80/86) and may cause its internalization through a process known as transendocytosis [89]. Tregs also induce the B7-H4 and B7-H receptor expression which determines the tolerogenic phenotype of monocyte-derived cells [90]. The interaction between the LAG-3 receptor expressed by Tregs and MHC-II expressed by monocyte-derived cells, inducing an inhibitory signal, has been described [91]. In respect to the role of the cell contact-mediated pathway, described in the literature, however, we consider that the effect of GB-MSCs is predominantly mediated by secretory factors. As stated therein before, our results have shown that GB-MSCs secrete IL-6, TGF*β*, CCL-2, PGE2, and sVEGF and the same cytokines are secreted by classical MSCs. Regarding MSCs, a major role associated with the generation of immunosuppressive myeloid cells is attributed to TGF*β* [92], PGE2 [93, 94], sVEGF [95], and CCL-2 [96]. These cytokines (independently or in complex) induce augmented secretion of IL-10 by both monocytes and monocyte-derived cells and foster their phenotype of immature dendritic cells with tolerogenic function. A pivotal role in the aforementioned processes plays IL-6. It leads to diminished expression of HLA-II and B7 complex, either directly operating with the STAT-3 system [87, 97] or inducing the IL-10 autocrine loop [98]. IL-6 may act in cooperation with other cytokines such as PGE2 and sVEGF [87]. Alternatively, the release of IL-6 is induced by the interaction between sHLA-G secreted by MSCs and ILT-4 expressed on the surface of monocyte-derived cells [91].

In conclusion, as far as we know, the current manuscript describes for the first time the immunosuppressive effects of GB-MSCs. Our *in vitro* experiments display that under the influence of GB-MSCs, the number of Tregs raises, that of Th17 decreases, and monocytes undergo changes in their cell surface expression. We realize some of the drawbacks and the limitations of the current study. It is too descriptive, perhaps, some speculations are excessive, and most of our results confirm a well-known evidence for the effects of MSCs. However, we consider that the description of these effects exerted by GB-MSCs from the viewpoint of describing another aspect of immune suppression, carried out by the tumor, could be a promising therapeutic target in the treatment of patients.

## Figures and Tables

**Table 1:** Secretion of cytokines (IL-6, IL-17A, IL-8, TGF*β*1, CCL-2, PGE2, and sVEGF-*α*) in culture media of GB-MSCs. The results are presented as the mean value obtained from ten GB-MSC cultures.

	pg/ml^∗^
IL-6	139.7 (90.3-187.6)
IL-8	1696.8 (1234.5-2010.4)
IL-17A	15.2 (12.7-37.2)
TGF*β*1	5100.0 (4950.0-5400.0)
CCL-2	3536.0 (3015.0-3873.5)
PGE_2_	1202.0 (980.4-1535.5)
sVEGF-*α*	4333.0 (4100.0-4750.0)

^∗^Data are presented as median (min-max).

## Data Availability

The data used to support the findings of this study are available from the corresponding author upon request.
